# Reconstruction of the regulatory hypermethylation network controlling hepatocellular carcinoma development during hepatitis C viral infection

**DOI:** 10.1515/jib-2023-0013

**Published:** 2023-11-20

**Authors:** Evgeniya A. Antropova, Tamara M. Khlebodarova, Pavel S. Demenkov, Anastasiia R. Volianskaia, Artur S. Venzel, Nikita V. Ivanisenko, Alexandr D. Gavrilenko, Timofey V. Ivanisenko, Anna V. Adamovskaya, Polina M. Revva, Nikolay A. Kolchanov, Inna N. Lavrik, Vladimir A. Ivanisenko

**Affiliations:** Institute of Cytology and Genetics, Siberian Branch of RAS, Novosibirsk, Russia; Kurchatov Genomic Center of the Institute of Cytology and Genetics of Siberian Branch of the Russian Academy of Sciences, Novosibirsk, Russia; Novosibirsk State University, Novosibirsk, Russia; Translational Inflammation Research, Medical Faculty, Otto von Guericke University Magdeburg, 39106 Magdeburg, Germany

**Keywords:** regulatory pathways, gene networks, ANDSystem software, differentially expressed genes, marker genes

## Abstract

Hepatocellular carcinoma (HCC) has been associated with hepatitis C viral (HCV) infection as a potential risk factor. Nonetheless, the precise genetic regulatory mechanisms triggered by the virus, leading to virus-induced hepatocarcinogenesis, remain unclear. We hypothesized that HCV proteins might modulate the activity of aberrantly methylated HCC genes through regulatory pathways. Virus-host regulatory pathways, interactions between proteins, gene expression, transport, and stability regulation, were reconstructed using the ANDSystem. Gene expression regulation was statistically significant. Gene network analysis identified four out of 70 HCC marker genes whose expression regulation by viral proteins may be associated with HCC: *DNA-binding protein inhibitor ID – 1 (ID1)*, *flap endonuclease 1 (FEN1)*, *cyclin-dependent kinase inhibitor 2A (CDKN2A)*, and *telomerase reverse transcriptase (TERT)*. It suggested the following viral protein effects in HCV/human protein heterocomplexes: HCV NS3(p70) protein activates human STAT3 and NOTC1; NS2-3(p23), NS5B(p68), NS1(E2), and core(p21) activate SETD2; NS5A inhibits SMYD3; and NS3 inhibits CCN2. Interestingly, NS3 and E1(gp32) activate c-Jun when it positively regulates *CDKN2A* and inhibit it when it represses *TERT*. The discovered regulatory mechanisms might be key areas of focus for creating medications and preventative therapies to decrease the likelihood of HCC development during HCV infection.

## 1 Introduction

Malignant diseases annually claim many lives worldwide. For instance, initial findings for 2021 suggest that cancer had ascended to being the top 2 leading cause of mortality in the USA [1]. HCC stands as one of the most frequently diagnosed cancers [2]. The hepatitis B and C viruses represent significant risk contributors to the development of HCC. Longitudinal research has demonstrated an elevated likelihood of HCC development within the population infected by HCV [3]. The prevalence of HCV infection among HCC patients was 44%–66 % in Italy [4, 5], while in Japan, it was as high as 80 % [6]. A meta-analysis of case-control investigations revealed that patients positive for anti-HCV have a 17-fold greater probability of developing HCC compared to those who tested negative for anti-HCV [7]. HCV actively controls host biological processes in infected cells whose dysfunction can provoke HCC development [8].

The mechanisms underlying HCV effects on host gene methylation have been widely discussed [9, 10]. Of great interest are studies related to HCV’s effect on the transcriptional regulation of genes subject to aberrant methylation in HCC due to their extreme importance for HCC development. Such data can shed light on virus-induced carcinogenesis mechanisms after infection of normal cells in which gene methylation is unimpaired. It can also identify genes whose expression after hypermethylation or hypomethylation can potentially be modulated by viral proteins. Identifying host-virus regulatory pathways can identify the viral proteins underlying HCC risk factors. Such data could be of great importance in the search for new drugs that target these viral proteins.

Computational of protein-protein interaction (PPI) networks analyses have found wide applications for establishing HCC molecular mechanisms. For example, methylated differentially expressed genes (DEGs) in HCC have been examined using PPI networks [11–16]. However, analyses based on reconstructed gene networks describing gene expression regulation remain to be performed to identify the regulatory mechanisms of genes important for HCC with participating viral proteins.

Reconstructing regulatory gene networks is broadly used to study the molecular mechanisms underlying diseases and biological processes [17–19]. In prior research, we established the ANDSystem software and information system designed to facilitate the reconstruction and analysis of gene networks. This system leverages automated knowledge extraction methods applied to the content of scientific literature and factographic databases [20–22]. Specifically, the ANDSystem has been employed in various studies, such as reconstructing and analyzing the pre-eclampsia associome [23] and HCV interactome [24]. It also assisted identifying of novel susceptibility candidates for tuberculosis [25] and the discovery of potential candidate genes integral to the comorbidity of asthma and hypertension [26]. Furthermore, the software has been used in the analysis of programmed cell death modulation during severe acute respiratory syndrome coronavirus 2 (SARS-CoV-2) infections [27], as well as in the identification of molecular mechanisms that involve nonstructural SARS-CoV-2 viral proteins in the metabolic dysregulation observed in SARSCoV-2-infected patients. This latter application was based on an analysis of plasma metabolomics and gene regulatory networks [28].

In this study, we employ the ANDSystem to reconstruct the regulatory pathways that may enable HCV proteins to modulate aberrantly methylated DEGs in HCC. The analysis examined 70 published HCC marker genes derived from genome-wide methylation analyses. Seven types of regulatory pathways that describe the influence of these genes by viral proteins were reconstructed. These pathways encompass PPIs, gene expression regulation, and the control of protein stability, activity, and transport. Among all the considered pathways, those involved in gene expression regulation were statistically significant. Out of 70 marker genes, 17 were differentially expressed in HCV-infected Huh 7.5 cells [29, 30]. Analysis of gene expression regulation pathways identified 7 out of 17 HCC marker genes potentially subject to regulation by HCV proteins, of which four had unidirectionally altered (upregulated) expression in HCC patients and HCV-infected Huh 7.5 cells: *cyclin-dependent kinase inhibitor 2A (CDKN2A), flap endonuclease 1 (FEN1), DNA-binding protein inhibitor ID – 1 (ID1)*, and *telomerase reverse transcriptase (TERT)*. Among those examined, we hypothesized that these genes might be responsible for the molecular mechanisms determining HCV as a risk factor for developing HCC.

Analyzing regulatory networks for these genes enabled us to make assumptions about viral protein effects on human protein functions in heterocomplexes formed due to PPIs between viral and human proteins in the considered networks. In particular, physical interactions between NS3 (p70) protein with human STAT3 cause its activation. NS3 can exert a similar effect on NOTC1 when heterocomplexed with it. Interactions between the protease NS5B (p68), envelope glycoprotein E2 (NS1), core viral (p21), and NS2-3 (p23) proteins with SETD2 are also expected to affect it positively. In contrast, interaction between viral proteins NS5A (p56) with human SMYD3 is expected to be inhibitory.

Of interest was the expected effect of interactions between viral protein NS3 (p70) and envelop glycoprotein E1 (gp32) with c-Jun. The network analysis indicated that NS3 and gp32 activate c-Jun when it positively regulates *CDKN2A* but inhibit it when it represses *TERT*.

Therefore, HCV protein modulation of host regulatory pathways may be important in HCC development during HCV infection. The findings of this study may provide valuable insights for the design of future experimental studies, such as the search for novel therapeutic targets or the development of pharmaceutical and prophylactic agents to mitigate the risk of HCC in the context of HCV infection.

## 2 Materials and methods

### 2.1 Analysis of genes exhibiting hypermethylation in HCC

Data on genes exhibiting aberrant methylation patterns in HCC were obtained from the scientific literature (Supplementary Table S1). Only those genes identified by the authors as the most significant were considered, creating a list of 70 genes. The largest gene groups were taken from a meta-analysis of differentially methylated genes [31] and an integrated analysis of microarray-based mRNA expression and methylation profiles [32]. Authors [31] used data on differential gene methylation in HCC tumor and adjacent tissue pairs, HCC tumor and normal tissue pairs, and HCC and normal serum pairs. They reported 22 marker genes, of which we excluded seven because their names did not allow us to accurately determine their identifiers in the Gene database: *p16*, *p14*, *CDH1*, *p15*, *hMLH1*, and *p73*. Ma et al. [32] identified novel aberrantly methylated DEGs using data from the GEO database: GSE19665 and GSE62232 for mRNA expression and GSE60753 for methylation. They identified 185 genes, including those downregulated and hypermethylated and those upregulated and hypomethylated. We took 35 genes for further analysis, which the authors listed among the central genes in their PPI network.

### 2.2 DEGs in HCV-infected cells

Two distinct sets of DEGs between mock-infected control and HCV-infected cells were created [1]: a compilation of 1844 significantly differentially expressed annotated genes identified in Huh 7.5 cells undergoing acute infection and proliferation at a 72-h mark [2, 30] a collection of 1886 genes derived from the analysis of the microarray-based dataset GSE66842 [29]. The GSE66842 dataset encompasses gene expression profiles of differentiated Huh7.5.1 cells subjected to infection with the HCV Jc1 clone. We used GEO2R [33] to screen for DEGs in 10-day post-infection samples in the dataset. We utilized an established threshold defined by a false discovery rate (FDR) below 0.05 to recognize differentially expressed genes.

### 2.3 Reconstructing regulatory pathways using the ANDSystem

Reconstruction of viral-protein-associated regulatory pathways containing HCC-associated genes (Table S1) was performed using the ANDSystem tool [21]. The ANDSystem knowledge base comprises a comprehensive gene network describing interactions between the objects of the ANDSystem’s ontology. This network was obtained via automated mining of scientific literature and factual databases. The ontology of the ANDSystem incorporates 13 distinct object types, including but not limited to proteins, genes, and metabolites, alongside 24 unique interaction types, such as expression, activity and stability regulation, physical interaction. Specific templates were used to search for pathways within the ANDSystem base of knowledge that dictate the structure of subgraphs embedded within the overarching gene network graph. Templates are defined as a sequential series of vertices that correspond to objects, where the edges that connect these vertices encapsulate the interactions between objects. Objects may be delineated either by a compilation of names or identifiers or, alternatively, by object type. In the first scenario, pathways containing objects from among those listed in the template will be searched. In the second case, any object of a given type can be included in the searched pathways.

We used seven different pathway templates (Table 1). The first object in all templates comprised HCV proteins, provided as a list of UniProt identifiers. The last object comprised a list of protein identifiers encoded by the genes in Table S1. The length of the templates varied from two to five objects. All intermediate objects were specified by their object type. Only human proteins and genes were considered intermediate objects. Interactions between objects in templates included PPIs, several regulatory interactions, and an expression-type relationship between a gene and its protein product. HCV proteins were only involved in PPIs with human proteins.

**Table 1:** Templates for virus-host interaction pathways.

*N*	Pathway template^a^
1.	Vp – PPI –> Tp
2.	Vp – PPI –> Hp – PPI –> Tp
3.	Vp – PPI –> Hp – Act/Stab/Pr/PPM/Tr –> Tp
4.	Vp – PPI –> Hp – Exp reg –> Tg – Exp –> Tp
5.	Vp – PPI –> Hp – Exp reg –> Hg – Exp –> Hp – Exp reg –> Tg – Exp –> Tp
6.	Vp – PPI –> Hp – Exp reg –> Hg – Exp –> Hp – Act/Stab/Pr/PPM/Tr –> Tp
7.	Vp – PPI –> Hp – Exp reg –> Hg – Exp –> Hp – PPI –> Tp

^a^Designations of objects and interactions: Vp, HCV proteins; Hp, any host proteins involved in the interactions; Hg, any host genes involved in the interactions; Tg, target gene (HCC marker gene); Tp, target protein (protein encoded by Tg); PPI, protein–protein interactions; Act/Stab/Pr/PPM/Tr, regulation of activity or stability or proteolysis or posttranslational modifications or transport (release); Exp reg, regulation of gene expression; Exp, gene expression (protein production).

## 3 Results

### 3.1 Identification of marker genes that can be regulated by viral proteins

We compiled a list of genes whose promoter hypermethylation or hypomethylation was accompanied by aberrant expression and associated with HCC in published data (Table S1). This list included 70 methylation-related DEGs. Most of these genes had been reported as HCC markers whose aberrant expression correlated with unfavorable disease prognoses. Regulatory pathways were reconstructed with the application of ANDSystem [21], intended to identify marker genes potentially regulated by viral proteins at the transcriptional regulation level, as illustrated in Table 1. We hypothesized that viral proteins could regulate such genes in normal and cancer cells, influencing the disease course and underlying risk factors. By deploying the ANDSystem software, the regulatory pathways that could potentially be modulated by HCV proteins influencing HCC marker genes were reassembled. This process was facilitated by templates that delineate various types of interactions between objects, as detailed in Table 2.

**Table 2:** Significance of virus–host interaction pathway templates.

Pathway template	Number of target genes	*P*-value	False discovery rate
1.	3	0.35	0.4
2.	40	0.06	0.105
3.	12	0.045	0.105
4.	**20**	**0.00017**	**0.0008**
5.	**51**	**0.00023**	**0.0008**
6.	24	0.17	0.238
7.	54	0.9	0.9

Values in bold were statistically significant (*p* < 0.0005).

In pathway templates, besides “expression regulation” interactions, we considered various interaction types, including PPIs; regulation of protein activity, stability, and transport (release); and post-translational modifications. Interestingly, among pathways involving all the regulation types mentioned above, only gene expression regulation represented by templates 4 and 5 was significant (Table 2). These pathways describe potential regulatory associations between HCV proteins and HCC marker genes with the participation of host mediator proteins and genes (Figures S1 and S2).

Nine HCV proteins, 19 host mediator proteins, and 20 HCC marker genes were involved in pathways associated with template 4 (Figures S1). Pathways reconstructed using template 5 contained 10 viral proteins, 51 HCC marker genes, 254 host mediator genes, and 344 host mediator proteins (Figures S2). According to the reconstructed regulatory pathways, HCV proteins possess the potential to modulate the expression of these hepatocellular carcinoma marker genes.

### 3.2 The employment of differential gene expression data derived from HCV-infected cell cultures

It should be noted that all reconstructed regulatory pathways are predictions and require further confirmation. In order to gain further insights, we analyzed previously published data on differential gene expression in HCV-infected cell cultures. Data concerning differential gene expression in Huh 7.5 cells acutely infected with HCV (72 h post-infection) were derived from the study by Papic et al. [30]. Additionally, another dataset, sourced from the work of Crouchet et al. [29] available in the GEO database (accession number GSE66842), included gene expression profiles of differentiated Huh 7.5.1 cells following infection with the HCV Jc1 clone. We used data representing 10 days post-infection in our analysis. In both studies, controls were mock-infected cells.

### 3.3 Reconstruction of regulatory pathways to differentially expressed genes in cell lines and patients

Of the 70 marker genes (Table S1), 17 were differentially expressed in HCV-infected cells (Table 3), of which seven were present in regulatory pathways (Figures S1 and S2). The data presented in Table 3 indicate a consistent pattern of alterations in gene expression among HCV-infected patients with HCC and corresponding cell lines. However, there are differences, which might reflect different aberrant methylation mechanisms in HCV presence and absence. The observed downregulation of tumor suppressor genes in patients, contrasted with their upregulation in HCV-infected cell lines, inversely mimicking the behavior of tumorigenic genes, suggests that not all regulatory pathways implicated in the transcriptional regulation of viral proteins necessarily contribute to the risk factors associated with HCC development. For further work, we considered only the gene group whose expression changed unidirectionally in patients and cell lines. Such genes may be of particular interest for studying the mechanisms through which HCV proteins influence HCC.

**Table 3:** Differentially expressed marker genes in HCV-infected cells.

Gene symbol	Crouchet et al. [29]		Papic et al. [30]		Expression in isolated	Expression in	Presence in regulatory
	Fold change	FDR	Fold change	FDR	infected cells^b^	HCC patients	pathways^c^
**CANX**	–	–	**1,6**	**921,475**	**Up**	**Up** [34]	**–**
**CDKN2A** ^**a**^	**2,74E−01**	^**5**^ **63E−02**	**–**	**–**	**Up**	**Up** [34, 35]	**4, 5**
**CEP55**	**3,51E−01**	**3,11E−02**	**–**	**–**	**Up**	**Up** [36]	**–**
**COL7A1**	**4,17E−01**	**1,69E−02**	**–**	**–**	**Up**	**Up** [37]	**–**
DSE	4,66E**−**01	3,13E**−**03	**–**	**–**	Up	Down [38]	**–**
**FAT1**	**5,10E−01**	**4,59E−03**	**–**	**–**	**Up**	**Up** [39]	**–**
**FEN1**	**5,81E−01**	**7,09E−03**	**–**	**–**	**Up**	**Up** [40]	**4, 5**
FNIP1	**–**	**–**	1,8	63,405	Up	Down [34]	**5**
**ID1**	**1,12**	**1,88E−04**	**2**	**445,256**	**Up**	**Up** [41]	**4, 5**
**MCM6**	**6,06E−01**	**3,10E−03**	**–**	**–**	**Up**	**Up** [42]	**–**
**NUSAP1**	**4,81E−01**	**1,30E−02**	**–**	**–**	**Up**	**Up** [43]	**–**
PROZ	−7,18E**−**01	5,85E**−**05	**–**	**–**	Down	Up [44]	**–**
RARB	2,23E**−**01	2,56E**−**02	**–**	**–**	Up	Down [32]	**4, 5**
RASSF1	6,49E**−**01	1,60E**−**04	2,1	75,092	Up	Down [34, 45]	**4, 5**
**RFC4**	**5,05E−01**	**5,84E−03**	**–**	**–**	**Up**	**Up** [46]	**–**
**RRAGD**	**1,04**	**1,96E−05**	**–**	**–**	**Up**	**Up** [47]	**–**
**TERT**	**–**	**–**	**1,8**	**20,886**	**Up**	**Up** [48]	**4, 5**

^a^The signifcance value slightly exceeds 0.05. However, the gene is considered because of its importance. ^b^Huh7.5.1 Huh7.5. ^c^The pathre specified by the number of pathway templates. Bold indicates genes whose expression changes coincide in patients and cell cultures.

### 3.4 Pathways with differentially expressed target marker and mediator genes

**Figure 1:**
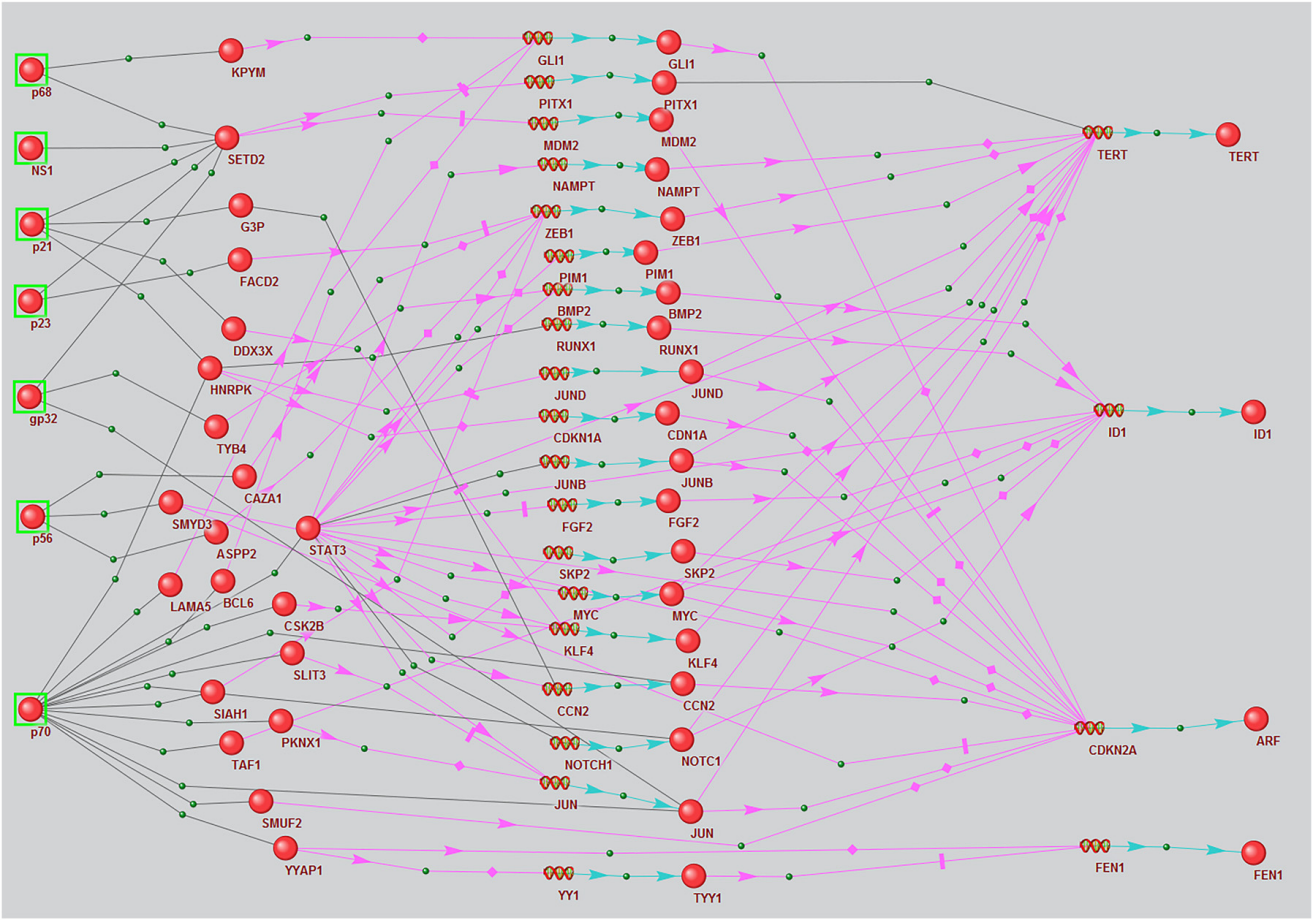
Regulatory pathways associated with viral protein modulation of the expression of HCC marker genes differentially expressed in infected cells, reconstructed using the ANDSystem according to combined templates 4 and 5. Balls with green frames denote viral proteins, other balls denote host proteins, spirals in the middle of the picture denote intermediate participant genes, and spirals on the right side denote marker genes. Black links between objects mean physical interaction, pink arrows – regulation of expression, blue arrows – production of a protein product from a gene.

The next step in our analysis was reconstructing regulatory pathways in which both target marker and mediator genes are differentially expressed in HCV-infected cell lines (Figure 1). Our assumption was predicated on the notion that alterations in gene expression can correlate with the functional activities of viral proteins implicated in these regulatory pathways. The requirement for differential expression did not apply to proteins involved in PPIs with HCV proteins since their expression might not depend on viral proteins.

### 3.5 *ID1*, *FEN1*, *TERT*, and *CDKN2A* regulatory pathways

Seven HCV proteins, 39 host mediator proteins, and four HCC marker genes are involved in pathways according to templates 4 and 5 (Figure 1). These marker genes were *ID1*, *FEN1*, *TERT*, and *CDKN2A*.

**Figure 2:**
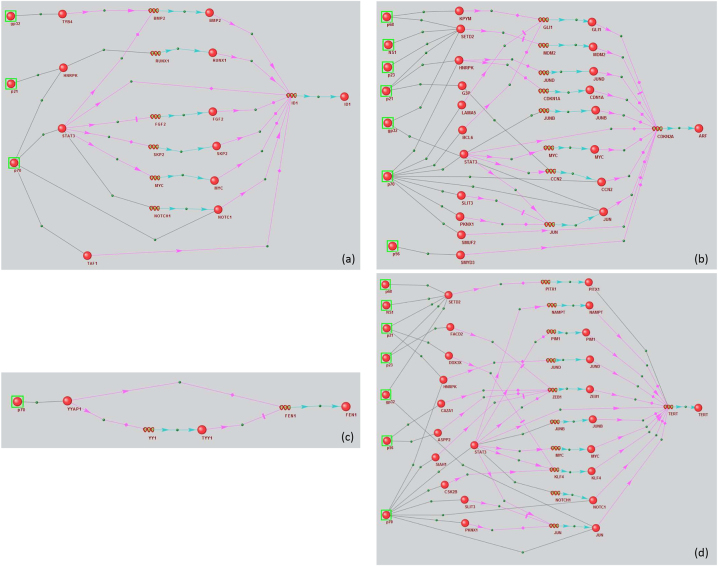
Regulatory networks of marker genes (a) ID1, (b) CDKN2A, (c) TERT, and (d) FEN1 modulated by HCV proteins. Black links between objects mean physical interaction, pink arrows – regulation of expression, blue arrows – production of a protein product from a gene.

We reviewed individual regulatory pathways for each gene (Figure 2a–d). Three viral proteins, 10 human proteins, and six human genes were involved in regulating *ID1* expression (Figure 2a). The viral proteins included gp32, core protein (p21), and NS3 (p70). NS3 had the most PPIs with human proteins.

Seven viral proteins, 19 human proteins, and eight human genes were involved in regulating *CDKN2A* (Figure 2b). The viral proteins included gp32, core protein (p21), NS5B (p68), protease NS2-3 (p23), NS5B (p56), NS1, and NS3 (p70).

The *FEN1* regulatory network (Figure 2c) contained only one viral NS3 protein (p70) and two intermediate participants (the human YYAP1 protein and *TYY1* gene and protein).

The TERT regulatory network involved seven viral proteins (gp32, p21, p23, p56, p68, p70, and NS1), 21 human proteins, and 10 human genes (Figure 2d). Among viral proteins, the NS3 protein had the most interactions with human proteins, consistent with the previous cases.

## 4 Discussion

The scientific literature has extensively discussed the regulation of host gene methylation by HCV proteins [9, 10]. Many genes subject to aberrant methylation contribute significantly to HCC pathophysiological mechanisms. Therefore, data on their transcriptional regulation, in which viral proteins are involved, may be of great interest. It is plausible to posit that viral protein regulatory interactions with these genes, potentially mediated through their transcription factors, could either repress or induce their expression. These effects could have similar consequences to hypermethylation or hypomethylation, provided HCV infection does not alter their methylation profile. In addition, viral regulation of the expression of already methylated genes can also change their expression profile. However, HCV’s effect on the transcriptional regulation of aberrantly methylated genes in HCC has not been systematically analyzed using reconstructed regulatory gene networks.

In this study, we employed the ANDSystem [21] to reconstruct and evaluate the statistical significance of regulatory pathways that potentially mediate the influence of hepatitis C virus proteins on host genes. We analyzed a set of previously reported genes aberrantly methylated in HCC patients in genome-wide methylation analyses (Table S1). This set included 70 genes previously characterized as epigenetic gatekeepers, HCC tumor suppressors, and protumorigenic factors.

Supplementing the data on anomalous methylation and gene expression sourced from patients, we incorporated data on differential gene expression in HCV-infected Huh 7.5 and 7.5.1 cells [29, 30]. Only 17 out of the 70 marker genes exhibited differential expression in HCV-infected cells.

It could be expected that viral proteins might regulate these genes through changes in their methylation status and effects on transcription factors. Indeed, 7 of the 17 DEGs were participants in regulatory pathways describing their expression regulation by viral proteins.

The virus-induced upregulation of tumorigenic gene expression and suppression of tumor suppressor gene expression in HCC patients may indicate the viral proteins’ contribution to HCC. Therefore, we considered the correspondence between the directions of marker gene expression changes in data for patients and cell cultures. A unidirectional change in gene expression in these datasets, activating tumorigenic genes or inhibiting tumor suppressor genes, suggests that they constitute one HCV pathological mechanism.

The next stage of the analysis was reconstructing regulatory networks with the requirement that all marker and mediator genes encoding transcription factors regulating marker genes show differential expression in HCV-infected cells (Figure 1). By incorporating this additional criterion, the count of marker genes within the regulatory network exhibiting unidirectional expression alterations in both HCC patients and cell lines was reduced to four: ID1, FEN1, CDKN2A, and TERT. We hypothesize that the influence of viral proteins on the expression of these genes might play a role in the mechanisms that render HCV a risk factor for HCC.

### 4.1 *ID1’s* regulatory network

ID1’s regulatory network is shown in Figure 2a. ID1 expression was upregulated in HCC patients and HCV-infected cells [41] (Table 3). There are three short pathways in the regulatory network that bind viral proteins to *ID1*; all involve NS3 (NS3/STAT3/*ID1*, NS3/NOTC1/*ID1*, and NS3/TAF1/*ID1*). While physical interaction between NS3 viral protein and STAT3 has been previously shown [49], their effects remain unclear. STAT3 is directly associated with ID1 upregulation [50]. In addition, STAT3 can regulate *ID1* by regulating *MYC* and *FGF2*, whose expression is upregulated by STAT3 [51, 52]. It should be noted that *MYC* and *FGF2* expression was elevated in HCV-infected Huh 7.5 cells [30]. Likewise, while NS3’s physical interaction with NOTC1 has been previously shown [49], its effects on NOTC1 function remain unclear. NS3 can also positively regulate NOTCH1 expression via STAT3. Data on physical interactions between STAT3 and NOTCH1 have been reported [53]. NOTCH1 then positively regulates *ID1* expression [54].

Therefore, an analysis of the regulatory network indicates that PPIs between viral NS3 with human STAT3 accompany ID1 activation. In addition, NS3’s interaction with NOTC1 also positively affects its activity.

### 4.2 *CDKN2A’s* regulatory network

*CDKN2A* expression was upregulated in HCC patients and HCV-infected cells (Table 3). Similar to ID1, NS3 (p70) occupies a central place in the network with respect to interaction numbers with human proteins (Figure 2b).

SETD2 was the human protein targeted by the most viral proteins, interacting with five: gp32, core protein (p21), NS5B (p68), NS2-3 (p23), and NS1. The regulatory network contains five short pathways linking viral proteins to *CDKN2A*: NS3/CCN2/*CDKN2A*, NS3/JUN/*CDKN2A*, NS3/STAT3/*CDKN2A*, NS3/SMUF2/*CDKN2A*, and NS5A(p56)/SMYD3/*CDKN2A*. Four of these pathways involve the viral protein NS3.

Published data has documented PPIs between NS5A and SMYD3 [49, 55, 56]. These studies demonstrated that NS5A co-localizes with SMYD3 solely in the cytoplasm, inhibiting SMYD3’s nuclear localization [55]. The capacity of SMYD3 to suppress CDKN2A expression has also been explored in the literature [57]. The regulatory network analysis indicated that NS5A’s interaction with SMYD3 inhibits SMYD3, suppressing its ability to inhibit *CDKN2A* expression, consistent with increased *CDKN2A* expression with HCV infection. Another *CDKN2A* activation pathway involves viral protein-mediated suppression of its inhibitors and may involve NS3/CCN2/*CDKN2A* interactions. For example, data on CCN2’s ability to negatively affect *CDKN2A* expression has been reported [58]. NS3 can physically interact with CCN2 [49]. Therefore, it can be hypothesized that NS3’s interaction with CCN2 might prevent CCN2 from inhibiting *CDKN2A* expression.

The NS3/STAT3/MYC/*CDKN2A* pathway might be the *CDKN2A* activation pathway resulting from the positive effects of viral proteins on human proteins. The NS3 protein’s effects on human gene expression have been previously discussed [59]. It should be noted that positive *CDKN2A* regulators, such as MYC, JUN, and GLI1, are upregulated in HCV-infected cells [29, 30].

Therefore, this regulatory network analysis supports the following assumptions about viral protein effects on human protein functions due to their physical PPIs: NS5A inhibits SMYD3, NS3 inhibits CCN2, and NS3, gp32 activates *c-Jun*.

### 4.3 *FEN1’s* regulatory network

FEN1 is controlled by the following participants: viral NS3 (p70), human YYAP1 and YY1 (p70; Figure 2c). While a physical interaction between NS3 and YYAP1 has been previously reported [49], the effects of PPIs between NS3 and YYAP1 on YYAP1 function have not been previously described. YYAP1 can negatively regulate *FEN1* expression by activating *YY1* expression, a *FEN1* transcriptional repressor [40, 60]. Interestingly, our reconstructed pathway indicates that increased *FEN1* expression with HCV infection can be explained by NS3 interacting with YYAP1, suppressing its ability to activate *YY1*.

### 4.4 *TERT’s* regulatory network

Like *ID1*, *FEN1*, and *CDKN2A*, *TERT* expression was upregulated in HCC patients and HCV-infected cells (Table 3). Four short pathways from viral proteins to *TERT* can be seen in the regulatory net-work: NS3(p70)/STAT3/*TERT*, NS3(p70)/gp32/JUN/*TERT*, p23/p68/NS1/p21/SETD2/*TERT*, and NS3/NOTC1/*TERT* (Figure 2d). Three participants in these pathways (STAT3, SETD2, and NOTC1) are positively associated with *TERT* [61–65], while JUN inhibits *TERT* expression [66, 67]. Since *TERT* was activated with HCV infection, it can be hypothesized that the physical interactions of viral proteins with positive *TERT* regulators STAT3, SETD2, and NOTC1 may positively affect their activity.

Physical interactions between c-JUN and viral proteins NS3 and E1 appeared to inhibit c-JUN. Interestingly, c-JUN was involved in *CDKN2A* upregulation in the *CDKN2A* regulatory network, and it was assumed that viral proteins activated c-JUN. However, c-JUN is inhibited in the *TERT* network, and it can be hypothesized that these same viral proteins interfere with its activity.

STAT3 was a member of the NS3/STAT3/*TERT* pathway and was associated with nine other nodes in the network. Therefore, it can be called the hub of this regulatory network. STAT3 activation by NS3 was discussed above for the ID1 regulatory network. The effects of NOTCH signaling pathway activation by the NS3 protein have been previously discussed [68]. However, it should again be noted that our data did not support the effect of NS3’s physical interaction with NOTC1. There is also no published information on the functional effects of SETD2’s physical interactions with viral proteins. Based on our analysis, SET2D’s interactions with viral proteins NS2-3 (p23), NS5B (p68), NS1, and NS3 (p70) might be expected to positively affect its ability to activate *TERT*.

Therefore, this regulatory network analysis is consistent with our assumption that the NS3 protein activates STAT3, and also activates NOTC1. Interestingly, the expected effects of NS3 and gp32 interaction with c-Jun are likely opposing when c-Jun acts as a *CDKN2A* activator and a *TERT* repressor.

We discussed the expected effects of physical interactions between viral NS3 protein and STAT3 in the *ID1*, *CDKN2A*, and *TERT* regulatory networks. We also hypothesized that viral NS3 interaction with STAT3 positively affects STAT3 activity for all three networks. Consequently, the reconstituted regulatory networks serve as a foundation for generating hypotheses about the molecular mechanisms underpinning HCV proteins’ impact on pivotal genes associated with HCC. Our qualitative analyses of these networks allowed us to propose several hypotheses about the mechanisms of host-virus interactions and the role of specific viral proteins in them. However, it should be noted that a rigorous quantitative analysis of the reconstructed networks requires mathematical modeling of their dynamics. Many interactions in gene networks can contribute non-linearly to the regulation of gene expression, the signal interference from opposing pathways can also have non-linear effects.

## Supplementary Material

Supplementary Material Details
