# Supplementary material for: Reconstruction of the regulatory hypermethylation network controlling hepatocellular carcinoma development during hepatitis C viral infection
Source: J Integr Bioinform. 2023 Nov 20;20(3):20230013. doi: 10.1515/jib-2023-0013 (PMC10757076; doi:10.1515/jib-2023-0013)
Supplement: Supplementary file 1 — Supplementary Material Details [file j_jib-2023-0013_suppl_001.docx]

**Supplementary materials**

**Reconstruction of the regulatory hypermethylation network controlling hepatocellular carcinoma development during hepatitis C viral infection** Antropova E.A., Khlebodarova T.M., Demenkov P.S., Volianskaia A.R., Venzel A.S., Ivanisenko N.V., Gavrilenko А.D., Ivanisenko T.V., Adamovskaya А.V., Revva P.M., Kolchanov N.A., Lavrik I.N. and Ivanisenko V.A.


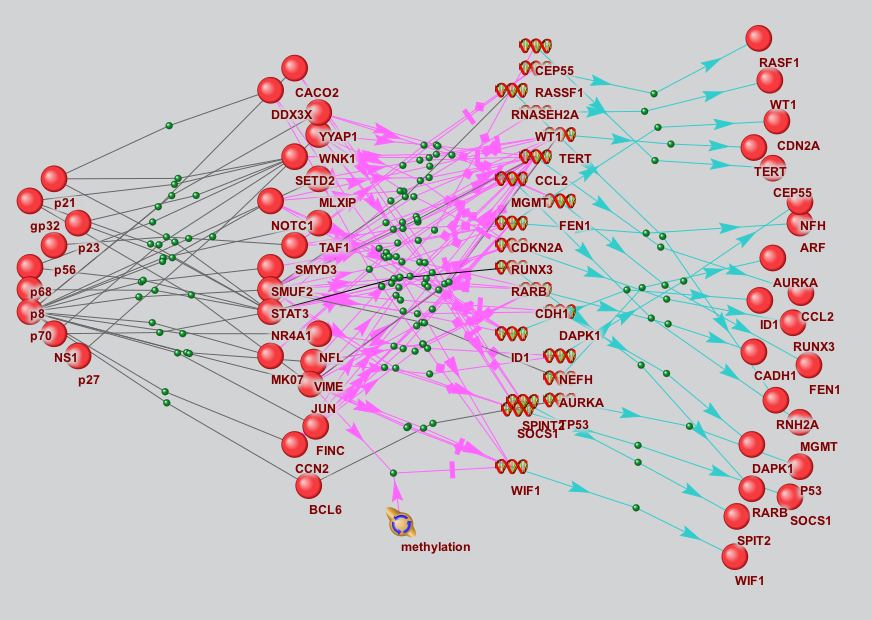


**Figure S1.** Regulatory pathways for modulation of the expression of HCC marker genes by viral proteins, built using ANDSystem according to template 3.


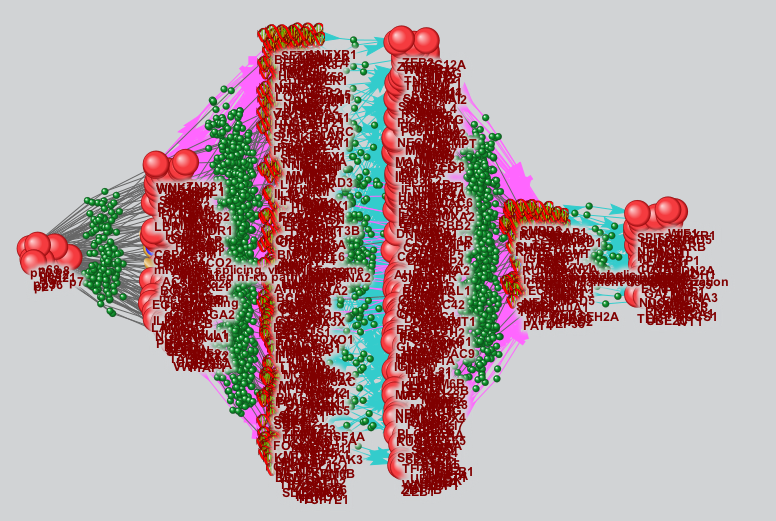


**Figure S2.** Regulatory pathways for modulation of the expression of HCC marker genes by viral proteins, built using ANDSystem according to template 4.

**Table S1.** The hypermethylated genes used in the analysis and their characterization in the literature.

| **Gene symbol** | **Protein name** | **Relation to tumorigenesis** | **Aberrant methylation information source** |
| --- | --- | --- | --- |
| *KCNA3* | Potassium voltage-gated channel subfamily A member 3 | Epigenetic gatekeeper candidates | [26] |
| *LDHB* | L-lactate dehydrogenase B chain |  |  |
| *SPINT2* | Kunitz-type protease inhibitor 2 |  |  |
| *TSPYL5* | Testis-specific Y-encoded-like protein 5 |  |  |
| *NEFH* | Neurofilament heavy polypeptide | Tumor suppressor genes in HCC [27] | [27] |
| *SMPD3* | Sphingomyelin phosphodiesterase 3 |  |  |
| *PER3* | Period circadian protein homolog 3 | Tumor suppressor [Hong Z, 2014] | [28] |
| *PROZ* | Vitamin K-dependent protein Z | Tumor suppressor [Neumann O, 2012] |  |
| *IGFALS* | Insulin-like growth-factor–binding protein, acid labile subunit | Tumor suppressor [Neumann O, 2012] |  |
| *TTC36* | Tetratricopeptide repeat protein 36 | Prognostic marker for predicting outcome and immune infiltration in HCC | [29] |
| *NEBL* | Nebulette | HCC specific diagnostic biomarkers |  |
| *FAM55C* | NXPE family member 3 |  | [30] |
| *GALNT3* | Polypeptide N-acetylgalactosaminyltransferase 3 |  |  |
| *DSE* | Dermatan-sulfate epimerase |  |  |
| *TERT* | Telomerase reverse transcriptase | Potential biomarker for HCC | [31] |
| *COL16A1* | Collagen alpha-1(XVI) chain | Mutational signatures and potential therapeutic targets | [32] |
| *COL7A1* | Collagen alpha-1(VII) chain |  |  |
| *FAT4* | Protocadherin Fat 4 |  |  |
| *RASSF1A* | Ras association domain-containing protein 1 | Tumor suppressor genes. DNA methylation biomarkers in HCC | [33] |
| *GSTP1* | Glutathione S-transferase P |  |  |
| *APC* | Adenomatous polyposis coli |  |  |
| *RUNX3* | Runt-related transcription factor 3 |  |  |
| *SOCS1* | Suppressor of the cytokine signalling 1 |  |  |
| MGMT | O6-methylguanine-DNA methyltransferase |  |  |
| *SFRP1* | Secreted frizzled-related protein 1 |  |  |
| *WIF1* | WNT inhibitory factor 1 |  |  |
| *PRDM2* | PR domain containing 2 |  |  |
| *DAPK1* | Death-associated protein kinase 1 |  |  |
| *p53* | Cellular tumor antigen p53 |  |  |
| *RARβ* | Retinoic acid receptor beta |  |  |
| *SPINT2* | Serine peptidase inhibitor, Kunitz type, 2 |  |  |
| *OPCML* | Opioid binding protein/cell adhesion molecule-like |  |  |
| *WT1* | Wilms tumor 1 |  |  |
| *ACADS* | Short-chain specific acyl-CoA dehydrogenase, mitochondrial | Oncogene [Chen D, 2019] | Ma, Z., (2019). Molecular Medicine Reports, 20(3), 2519-2532. |
| *APOF* | Apolipoprotein F | Tumor suppressor [Wang YB, 2019] |  |
| *ASS1* | Argininosuccinate synthase | Tumor suppressor [Huang HY] |  |
| *ATAD2* | ATPase family AAA domain-containing protein 2 | Oncogene [Hussain M, 2018] |  |
| *AURKA* | Aurora kinase A | Oncogene [Du R, 2021] |  |
| *CANX* | Calnexin | - |  |
| *CBFA2T3* | Protein CBFA2T3 | Tumor suppressor [Salah Z, 2015] |  |
| *CCL2* | C-C motif chemokine 2 | Has both anti-tumor and pro-tumor effects, depending on the interaction between cancer cells and host cells [Jin J, 2021] |  |
| *CDKN3* | Cyclin-dependent kinase inhibitor 3 | Tumor suppressor [Nalepa G, 2013] |  |
| *CEP55* | Centrosomal protein of 55 kDa | Oncogene [Li M, 2018] |  |
| *CTNNA1* | Catenin alpha-1 | Tumor suppressor [Hemmat M,2014] |  |
| *ECM1* | Extracellular matrix protein 1 | Tumor suppressor [Gao F, 2014] |  |
| *FAT1* | Protocadherin Fat 1 | Oncogene [Valletta D, 2014] |  |
| *FEN1* | Flap endonuclease 1 | Oncogene [Zhang Y, 2020] |  |
| *FNIP1* | Folliculin-interacting protein 1 | Tumor suppressor [Hasumi H, 2015] |  |
| *FOS* | Protein c-Fos | Tumor suppressor [Mikula M, 2003] |  |
| *ID1* | DNA-binding protein inhibitor ID-1 | Oncogene [Yin X, 2017] |  |
| *IGFBP3* | Insulin-like growth factor-binding protein 3 | Tumor suppressor [Law CT, 2019] |  |
| *KIF4A* | Chromosome-associated kinesin KIF4A | Oncogene [Hou G, 2017] |  |
| *LAMC1* | Laminin subunit gamma-1 | Oncogene [Mou Y, 2022] |  |
| *LPL* | Lipoprotein lipase | Tumor suppressor [Mithani SK, 2011] |  |
| *MCM4* | DNA replication licensing factor MCM4 | Oncogene [Jia M, 2022] |  |
| *MCM6* | DNA replication licensing factor MCM6 | Oncogene [Wang Y, 2022] |  |
| *NDC80* | Kinetochore protein NDC80 homolog | Oncogene [Chen X, 2022] |  |
| *NUSAP1* | Nucleolar and spindle-associated protein 1 | Oncogene [Hu Y, 2022] |  |
| *RASSF1* | Ras association domain-containing protein 1 | Tumor suppressor [Li W, 2019] |  |
| *RFC4* | Replication factor C subunit 4 | Oncogene [Arai M, 2009] |  |
| *RNASEH2A* | Ribonuclease H2 subunit A | Oncogene [Zhao F, 2022] |  |
| *RRAGD* | Ras-related GTP-binding protein D | Oncogene [Wang G, 2022] |  |
| *SHBG* | Sex hormone-binding globulin | Tumor suppressor [Kahn SM, 2008] |  |
| *SLC10A1* | Hepatic sodium/bile acid cotransporter | Tumor suppressor [Lu C, 2020] |  |
| *SLC22A1* | Solute carrier family 22 member 1 | Tumor suppressor [Lautem A, 2013] |  |
| *SMAD5* | Mothers against decapentaplegic homolog 5 | Oncogene [Li S, 2019] |  |
| *TXNRD1* | Thioredoxin reductase 1, cytoplasmic | Oncogene [Huang WY, 2022] |  |
| *UBE2C* | Ubiquitin-conjugating enzyme E2 C | Oncogene [Pan W, 200] |  |
| *WFDC1* | WAP four-disulfide core domain protein 1 | Tumor suppressor [Liu S, 2009] |  |
| *CDKN2A* | CDKN2A-interacting protein | Tumor suppressor [Zhao R., 2016] | Feng et al., 2010 |
| *CDH1* | Cadherin-1 | Tumour suppressor | El-Bendary et al., 2020 |

**References**

1. Hong Z, Feng Z, Sai Z, Tao S. PER3, a novel target of miR-103, plays a suppressive role in colorectal cancer in vitro. BMB Rep. 2014 Sep;47(9):500-5. doi: 10.5483/bmbrep.2014.47.9.212.
2. Neumann O, Kesselmeier M, Geffers R, Pellegrino R, Radlwimmer B, Hoffmann K, Ehemann V, Schemmer P, Schirmacher P, Lorenzo Bermejo J, Longerich T. Methylome analysis and integrative profiling of human HCCs identify novel protumorigenic factors. Hepatology. 2012 Nov;56(5):1817-27. doi: 10.1002/hep.25870.
3. Feng Q, Stern JE, Hawes SE, Lu H, Jiang M, Kiviat NB. DNA methylation changes in normal liver tissues and hepatocellular carcinoma with different viral infection. Exp Mol Pathol. 2010;88:287–292.
4. El-Bendary M, Nour D, Arafa M, Neamatallah M. Methylation of tumour suppressor genes RUNX3, RASSF1A and E-Cadherin in HCV-related liver cirrhosis and hepatocellular carcinoma. Br J Biomed Sci. 2020 Jan;77(1):35-40. doi: 10.1080/09674845.2019.1694123.
5. Chen D, Feng X, Lv Z, Xu X, Lu Y, Wu W, Wu H, Liu H, Cao L, Ye S, Chen J, Wu J. ACADS acts as a potential methylation biomarker associated with the proliferation and metastasis of hepatocellular carcinomas. Aging (Albany NY). 2019 Oct 25;11(20):8825-8844. doi: 10.18632/aging.102292
6. Wang YB, Zhou BX, Ling YB, Xiong ZY, Li RX, Zhong YS, Xu MX, Lu Y, Liang H, Chen GH, Yao ZC, Deng MH. Decreased expression of ApoF associates with poor prognosis in human hepatocellular carcinoma. Gastroenterol Rep (Oxf). 2019 Apr 21;7(5):354-360. doi: 10.1093/gastro/goz011.
7. Huang HY, Wu WR, Wang YH, Wang JW, Fang FM, Tsai JW, Li SH, Hung HC, Yu SC, Lan J, Shiue YL, Hsing CH, Chen LT, Li CF. ASS1 as a novel tumor suppressor gene in myxofibrosarcomas: aberrant loss via epigenetic DNA methylation confers aggressive phenotypes, negative prognostic impact, and therapeutic relevance. Clin Cancer Res. 2013 Jun 1;19(11):2861-72. doi: 10.1158/1078-0432.CCR-12-2641.
8. Hussain M, Zhou Y, Song Y, Hameed HMA, Jiang H, Tu Y, Zhang J. ATAD2 in cancer: a pharmacologically challenging but tractable target. Expert Opin Ther Targets. 2018 Jan;22(1):85-96. doi: 10.1080/14728222.2018.1406921.
9. Du R, Huang C, Liu K, Li X, Dong Z. Targeting AURKA in Cancer: molecular mechanisms and opportunities for Cancer therapy. Mol Cancer. 2021 Jan 15;20(1):15. doi: 10.1186/s12943-020-01305-3.
10. Salah Z, Arafeh R, Maximov V, Galasso M, Khawaled S, Abou-Sharieha S, Volinia S, Jones KB, Croce CM, Aqeilan RI. miR-27a and miR-27a* contribute to metastatic properties of osteosarcoma cells. Oncotarget. 2015 Mar 10;6(7):4920-35. doi: 10.18632/oncotarget.3025.
11. Jin J, Lin J, Xu A, Lou J, Qian C, Li X, Wang Y, Yu W, Tao H. CCL2: An Important Mediator Between Tumor Cells and Host Cells in Tumor Microenvironment. Front Oncol. 2021 Jul 27;11:722916. doi: 10.3389/fonc.2021.722916.
12. Nalepa G, Barnholtz-Sloan J, Enzor R, Dey D, He Y, Gehlhausen JR, Lehmann AS, Park SJ, Yang Y, Yang X, Chen S, Guan X, Chen Y, Renbarger J, Yang FC, Parada LF, Clapp W. The tumor suppressor CDKN3 controls mitosis. J Cell Biol. 2013 Jun 24;201(7):997-1012. doi: 10.1083/jcb.201205125.
13. Li M, Gao J, Li D, Yin Y. CEP55 Promotes Cell Motility via JAK2⁻STAT3⁻MMPs Cascade in Hepatocellular Carcinoma. Cells. 2018 Aug 8;7(8):99. doi: 10.3390/cells7080099.
14. Hemmat M, Chen W, Anguiano A, Naggar ME, Racke FK, Jones D, Wang Y, Strom CM, Chang K, Boyar FZ. Submicroscopic deletion of 5q involving tumor suppressor genes (CTNNA1, HSPA9) and copy neutral loss of heterozygosity associated with TET2 and EZH2 mutations in a case of MDS with normal chromosome and FISH results. Mol Cytogenet. 2014 May 27;7:35. doi: 10.1186/1755-8166-7-35.
15. Gao F, Xia Y, Wang J, Lin Z, Ou Y, Liu X, Liu W, Zhou B, Luo H, Zhou B, Wen B, Zhang X, Huang J. Integrated analyses of DNA methylation and hydroxymethylation reveal tumor suppressive roles of ECM1, ATF5, and EOMES in human hepatocellular carcinoma. Genome Biol. 2014 Dec 3;15(12):533. doi: 10.1186/s13059-014-0533-9.
16. Valletta D, Czech B, Spruss T, Ikenberg K, Wild P, Hartmann A, Weiss TS, Oefner PJ, Müller M, Bosserhoff AK, Hellerbrand C. Regulation and function of the atypical cadherin FAT1 in hepatocellular carcinoma. Carcinogenesis. 2014 Jun;35(6):1407-15. doi: 10.1093/carcin/bgu054.
17. Zhang Y, Liu X, Liu L, Chen J, Hu Q, Shen S, Zhou Y, Chen S, Xue C, Cui G, Yu Z. Upregulation of FEN1 Is Associated with the Tumor Progression and Prognosis of Hepatocellular Carcinoma. Dis Markers. 2020 Jan 13;2020:2514090. doi: 10.1155/2020/2514090.
18. Hasumi H, Baba M, Hasumi Y, Lang M, Huang Y, Oh HF, Matsuo M, Merino MJ, Yao M, Ito Y, Furuya M, Iribe Y, Kodama T, Southon E, Tessarollo L, Nagashima K, Haines DC, Linehan WM, Schmidt LS. Folliculin-interacting proteins Fnip1 and Fnip2 play critical roles in kidney tumor suppression in cooperation with Flcn. Proc Natl Acad Sci U S A. 2015 Mar 31;112(13):E1624-31. doi: 10.1073/pnas.1419502112.
19. Mikula M, Gotzmann J, Fischer AN, Wolschek MF, Thallinger C, Schulte-Hermann R, Beug H, Mikulits W. The proto-oncoprotein c-Fos negatively regulates hepatocellular tumorigenesis. Oncogene. 2003 Oct 2;22(43):6725-38. doi: 10.1038/sj.onc.1206781.
20. Yin X, Tang B, Li JH, Wang Y, Zhang L, Xie XY, Zhang BH, Qiu SJ, Wu WZ, Ren ZG. ID1 promotes hepatocellular carcinoma proliferation and confers chemoresistance to oxaliplatin by activating pentose phosphate pathway. J Exp Clin Cancer Res. 2017 Nov 23;36(1):166. doi: 10.1186/s13046-017-0637-7.
21. Law CT, Wei L, Tsang FH, Chan CY, Xu IM, Lai RK, Ho DW, Lee JM, Wong CC, Ng IO, Wong CM. HELLS Regulates Chromatin Remodeling and Epigenetic Silencing of Multiple Tumor Suppressor Genes in Human Hepatocellular Carcinoma. Hepatology. 2019 May;69(5):2013-2030. doi: 10.1002/hep.30414.
22. Hou G, Dong C, Dong Z, Liu G, Xu H, Chen L, Liu L, Wang H, Zhou W. Upregulate KIF4A Enhances Proliferation, Invasion of Hepatocellular Carcinoma and Indicates poor prognosis Across Human Cancer Types. Sci Rep. 2017 Jun 23;7(1):4148. doi: 10.1038/s41598-017-04176-9.
23. Mou Y, Sun Q. The long non-coding RNA ASMTL-AS1 promotes hepatocellular carcinoma progression by sponging miR-1343-3p that suppresses LAMC1 (laminin subunit gamma 1). Bioengineered. 2022 Jan;13(1):746-758. doi: 10.1080/21655979.2021.2012628.
24. Mithani SK, Smith IM, Califano JA. Use of integrative epigenetic and cytogenetic analyses to identify novel tumor-suppressor genes in malignant melanoma. Melanoma Res. 2011 Aug;21(4):298-307. doi: 10.1097/CMR.0b013e328344a003.
25. Jia M, Feng S, Cao F, Deng J, Li W, Zhou W, Liu X, Bai W. Identification of EGFR-Related LINC00460/mir-338-3p/MCM4 Regulatory Axis as Diagnostic and Prognostic Biomarker of Lung Adenocarcinoma Based on Comprehensive Bioinformatics Analysis and Experimental Validation. Cancers (Basel). 2022 Oct 17;14(20):5073. doi: 10.3390/cancers14205073.
26. Wang Y, Chen H, Liu W, Yan H, Zhang Y, Cheung AHK, Zhang J, Chen B, Liang L, Zhou Z, Wong CC, Wu WKK, Chan MWY, Cheng ASL, Ma BBY, Yu J, Lo KW, To KF, Kang W. MCM6 is a critical transcriptional target of YAP to promote gastric tumorigenesis and serves as a therapeutic target. Theranostics. 2022 Sep 6;12(15):6509-6526. doi: 10.7150/thno.75431.
27. Chen X, He Q, Zeng S, Xu Z. Upregulation of nuclear division cycle 80 contributes to therapeutic resistance via the promotion of autophagy-related protein-7-dependent autophagy in lung cancer. Front Pharmacol. 2022 Aug 29;13:985601. doi: 10.3389/fphar.2022.985601.
28. Hu Y, Xue Z, Qiu C, Feng Z, Qi Q, Wang J, Jin W, Zhong Z, Liu X, Li W, Zhang Q, Huang B, Chen A, Wang J, Yang N, Zhou W. Knockdown of NUSAP1 inhibits cell proliferation and invasion through downregulation of TOP2A in human glioblastoma. Cell Cycle. 2022 Sep;21(17):1842-1855. doi: 10.1080/15384101.2022.2074199.
29. Li W, Yue F, Dai Y, Shi B, Xu G, Jiang X, Zhou X, Pfeifer GP, Liu L. Suppressor of hepatocellular carcinoma RASSF1A activates autophagy initiation and maturation. Cell Death Differ. 2019 Aug;26(8):1379-1395. doi: 10.1038/s41418-018-0211-7.
30. Arai M, Kondoh N, Imazeki N, Hada A, Hatsuse K, Matsubara O, Yamamoto M. The knockdown of endogenous replication factor C4 decreases the growth and enhances the chemosensitivity of hepatocellular carcinoma cells. Liver Int. 2009 Jan;29(1):55-62. doi: 10.1111/j.1478-3231.2008.01792.x.
31. Zhao F, Liu A, Gong X, Chen H, Wei J, Chen B, Chen S, Yang R, Fan Y, Mao R. Hypoxia-induced RNASEH2A limits activation of cGAS-STING signaling in HCC and predicts poor prognosis. Tumori. 2022 Feb;108(1):63-76. doi: 10.1177/03008916211026019.
32. Wang G, Lu Y, Di S, Xie M, Jing F, Dai X. miR-99a-5p inhibits glycolysis and induces cell apoptosis in cervical cancer by targeting RRAGD. Oncol Lett. 2022 May 27;24(1):228. doi: 10.3892/ol.2022.13349.
33. Lautem A, Heise M, Gräsel A, Hoppe-Lotichius M, Weiler N, Foltys D, Knapstein J, Schattenberg JM, Schad A, Zimmermann A, Otto G, Lang H, Galle PR, Schuchmann M, Zimmermann T. Downregulation of organic cation transporter 1 (SLC22A1) is associated with tumor progression and reduced patient survival in human cholangiocellular carcinoma. Int J Oncol. 2013 Apr;42(4):1297-304. doi: 10.3892/ijo.2013.1840.
34. Li S, Zhao B, Zhao H, Shang C, Zhang M, Xiong X, et al. Silencing of long non-coding RNA SMAD5-AS1 reverses epithelial mesenchymal transition in nasopharyngeal Carcinoma via microRNA-195-dependent inhibition of SMAD5. Front Oncol 2019; 9:1246.
35. Huang WY, Liao ZB, Zhang JC, Zhang X, Zhang HW, Liang HF, Zhang ZY, Yang T, Yu J, Dong KS. USF2-mediated upregulation of TXNRD1 contributes to hepatocellular carcinoma progression by activating Akt/mTOR signaling. Cell Death Dis. 2022 Nov 1;13(11):917. doi: 10.1038/s41419-022-05363-x.
36. Pan W, Chen KJ, Huang YC. Ceramide synthase 6 antisense RNA 1 contributes to the progression of breast cancer by sponging miR-16-5p to upregulate ubiquitin-conjugating enzyme E2C. Anticancer Drugs. 2022 Oct 1;33(9):913-922. doi: 10.1097/CAD.0000000000001381.
37. Liu S, Howell P, Ren S, Fodstad O, Zhang G, Samant R, Shevde L, Xi Y, Pannell LK, Riker AI. Expression and functional analysis of the WAP four disulfide core domain 1 gene in human melanoma. Clin Exp Metastasis. 2009;26(7):739-49. doi: 10.1007/s10585-009-9273-8.
38. Zhao R., Choi B.Y., Lee M.H., Bode A.M., Dong Z. Implications of genetic and epigenetic alterations of CDKN2A (p16(INK4a)) in cancer. EBioMedicine 8, 30–39 (2016).
39. Kahn SM, Li YH, Hryb DJ, Nakhla AM, Romas NA, Cheong J, Rosner W. Sex hormone-binding globulin influences gene expression of LNCaP and MCF-7 cells in response to androgen and estrogen treatment. Adv Exp Med Biol. 2008;617:557-64. doi: 10.1007/978-0-387-69080-3_57.
40. Lu C, Fang S, Weng Q, Lv X, Meng M, Zhu J, Zheng L, Hu Y, Gao Y, Wu X, Mao J, Tang B, Zhao Z, Huang L, Ji J. Integrated analysis reveals critical glycolytic regulators in hepatocellular carcinoma. Cell Commun Signal. 2020 Jun 23;18(1):97. doi: 10.1186/s12964-020-00539-4.
